# Accessory–vagal crosstalk at the jugular foramen: a hypothesis for reflex dry cough during posterior cervical extracorporeal shockwave therapy

**DOI:** 10.3389/fresc.2026.1915218

**Published:** 2026-09-23

**Authors:** Xuewen Jia

**Affiliations:** Department of Joint Surgery and Sports Medicine, The First Affiliated Hospital of Ningbo University, Ningbo, Zhejiang, China

**Keywords:** accessory nerve, cough reflex, ephaptic transmission, extracorporeal shock wave therapy, jugular foramen, neural crosstalk, nucleus tractus solitarius, vagus nerve

## Abstract

**Background:**

Extracorporeal shockwave therapy (ESWT) is a core modality for treating myofascial pain and degenerative cervical spine conditions in the nuchal and back regions. However, a subset of patients clinically experiences sudden, transient, and irritative dry coughs when receiving treatment near the seventh cervical vertebra (C7, the Dazhui acupoint region). Traditional perspectives vaguely attribute this to “physical acoustic waves penetrating and vibrating the lung parenchyma,” which has caused excessive panic regarding iatrogenic pneumothorax and limited the rational titration of treatment intensity.

**Objective:**

This perspective aims to refute the traditional “lung vibration theory” through quantitative acoustic physics deductions and, for the first time, constructs a novel cross-system “Accessory-Vagal Crosstalk at the Jugular Foramen” hypothesis by integrating recent advancements in microanatomy.

**Mechanism:**

Recent anatomical studies confirm that the main trunk and dense branches of the accessory nerve (CN XI), which innervate the upper trapezius, are highly concentrated near C7. High-frequency and high-pressure mechanical shear forces released by ESWT strongly irritate the accessory nerve, detonating retrograde conduction of action potentials in the periphery. When this explosive electrical signal ascends to the narrow jugular foramen at the skull base, it breaches the myelin insulation barrier between nerve fibers and erroneously activates the closely aligned sensory fibers of the vagus nerve (CN X) and glossopharyngeal nerve (CN IX) through ephaptic transmission. This leaked electrical signal ascends into the nucleus tractus solitarius (NTS) in the medulla, which the central nervous system instinctively misinterprets as a high-level airway foreign body intrusion, thereby forcibly initiating a reflex dry cough.

**Clinical significance:**

This reflex exhibits a distinct “Instantaneous On-Off Effect”. In clinical practice, adjusting the applicator angle to a 15° to 30° tilt toward the vertebral body (to avoid the main accessory nerve plexus) and implementing a low-energy gradient “stepwise titration” can completely block this cross-system abnormal reflex while ensuring safety.

## 1 Introduction

Extracorporeal Shockwave Therapy (ESWT), as a non-invasive and highly effective physical therapy, has been widely applied in orthopedics, sports medicine, and pain rehabilitation in recent years (1, 2). By utilizing instantaneously generated high-pressure, high-frequency mechanical acoustic waves to penetrate soft tissues, it promotes local microcirculation, inhibits the release of inflammatory factors, and modulates pain transmission in free nerve endings via mechanotransduction. It has been clinically recognized as an “electronic scalpel” for treating Myofascial Pain Syndrome (MPS), nuchal ligament calcification, and degenerative cervical spondylosis (3).

In clinical practice, the vicinity of the C7 spinous process (the “Dazhui acupoint” area in traditional medicine) is a high-incidence area for nuchal/back myofascial pain and cervicobrachial syndrome, making it a core target for ESWT operations. However, a common phenomenon observed on the clinical frontline—yet rarely reported systematically in mainstream literature—is that some patients experience a sudden, transient, irritative dry cough when receiving shockwave treatment (especially focused shockwaves) in the C7 and adjacent areas. Once the impacts stop or the applicator angle is fine-tuned, the cough symptom typically ceases abruptly within milliseconds. Because this anatomical region is adjacent to the pulmonary apex, clinical peers domestically and internationally generally tend to characterize this as a physical stimulation response caused by “mechanical waves penetrating deep tissues, directly touching or vibrating the lung parenchyma and parietal pleura.” This intuitive, traditional explanation has provoked undue fear among frontline physical therapists regarding “iatrogenic pneumothorax” or pulmonary “barotrauma” (4), even leading some practitioners to blindly lower treatment energy or avoid this core target entirely, which severely limits the clinical efficacy of the shockwaves.

This perspective points out that the traditional “physical lung impact theory” possesses significant logical limitations in both acoustic physics and clinical presentation. Integrating the latest precise anatomical evidence of the trapezius muscle, we propose for the first time a novel cross-system “Accessory-Vagal Crosstalk at the Jugular Foramen” hypothesis. We posit that the essence of this clinical phenomenon is not organic damage to lung tissue. Rather, the high-intensity mechanical shear stress emitted by ESWT strongly irritates the dense main trunk of the accessory nerve (CN XI) within the upper trapezius, causing electrical signals to travel retrogradely along the axon up to the skull base. Within the narrow jugular foramen, the explosive electrical potential breaks through the insulation barrier, erroneously activating the closely adjacent sensory fibers of the vagus nerve (CN X) via ephaptic transmission. The leaked signal ultimately ascends to the nucleus tractus solitarius (NTS) in the medulla, causing the central nervous system to misinterpret the signal and forcibly trigger the “alarm” of a reflex dry cough.

Elucidating this mechanism not only fundamentally clarifies the clinical differential diagnosis points between “transient dry cough” and “iatrogenic pneumothorax” during shockwave therapy, eliminating unnecessary medical panic, but also provides a brand-new theoretical perspective for iatrogenic neuromodulation mechanisms in physical therapy.

## 2 Biomechanical fallacy: the “pulmonary apex barrier” mechanism based on acoustic impedance mismatch

In the clinical practice of ESWT, the “physical lung contact theory” regarding operations in the C7 region inducing irritative dry cough presents a fatal theoretical flaw based on fundamental biomedical acoustics. As a high-energy acoustic mechanical wave, the propagation, interfacial behavior, and energy release of shockwaves in the human body's composite media strictly adhere to acoustic impedance matching and acoustic dynamic laws.

### 2.1 Cliff-like mismatch of biological media acoustic impedance

When sound waves propagate through a medium, the medium's resistance to acoustic mechanical vibration conduction is called acoustic impedance (Z). Its basic physical formula is (5):

Z=ρ⋅c

The soft tissues of the human nuchal and back regions, including skin, subcutaneous fat, trapezius, nuchal ligament, and deep myofascia, have a physical density highly similar to water (ρ≈1000) and a sound velocity of approximately 1500m/s. Therefore, the apparent acoustic impedance of soft tissues ($Z_{soft}$) is approximately 1.5×10Pa⋅s/m (or 1.5 Mrayl).

However, when the shockwave attempts to cross the parietal pleura from deep soft tissues and transmit inward into the lung parenchyma, the medium changes to an air-rich alveolar structure. The physical density of gas is extremely low ($\rho \approx 1.2kg/m^{3)}$), with a sound velocity of about 340m/s, resulting in an acoustic impedance ($Z_{air}$) of only about 400Pa⋅s/m (approximately $4\times 10^{-4}Mrayl$)^[9]^. This indicates that there is a severe acoustic impedance mismatch region spanning several orders of magnitude across the pleural anatomical interface.

### 2.2 Near-total energy reflection at the “soft tissue-Gas” interface

According to classical wave acoustics, when a sound wave hits the interface of two media with different acoustic impedances perpendicularly or near-perpendicularly, the energy reflection coefficient (R, the ratio of reflected wave energy to incident wave energy) is determined by the absolute difference in acoustic impedance between the two media, calculated as:

$$R={\left(\frac{Z_{2}-Z_{1}}{Z_{2}+Z_{1}}\right)}^{2}$$

Substituting the soft tissue acoustic impedance $Z_{1}\approx 1.5\times 10^{6}Pa\cdot s/m$ and the intra-pulmonary gas acoustic impedance $Z_{2}\approx 400Pa\cdot s/m$ into the formula for quantitative analysis yields:

$$R={\left(\frac{400-1.5\times 10^{6}}{400+1.5\times 10^{6}}\right)}^{2}\approx {\left(\frac{-1499600}{1500400}\right)}^{2}\approx 0.9989$$

Quantitative calculations show that up to 99.9% of the shockwave's acoustic energy is reflected upon impacting the pleural and alveolar gas-liquid interface, with less than 0.1% of an extremely weak transmitted wave being able to cross the pleural barrier into the lung parenchyma. This “acoustic insulation wall,” naturally built by physical acoustic laws, means that the mechanical kinetic energy of exogenous shockwaves is physically truncated peripherally before it can touch the lung parenchyma.

### 2.3 Clinical counter-evidence of cavitation effect and barotrauma

The physical waveform of ESWT consists of a broadband positive high-pressure wave with an extremely steep pressure front (reaching tens of megapascals within nanoseconds) followed by a negative tensile wave (3). When the negative tensile wave propagates in liquid or high-density soft tissue media, it easily induces a strong cavitation effect, generating microsecond-level non-linear bubble growth and collapse, and releasing local microjets (5).

If we assume that the high-energy shockwaves during the procedure could defy acoustic impedance mismatch laws and “forcibly” transmit through the pleural barrier to act on lung tissue, the immense shear stress released during scattering and total reflection at the discontinuous interface would instantly cause alveolar wall rupture and microvascular network tearing (6). This is because the alveolar walls are composed of extremely thin single-layer epithelial cells and capillary endothelium, making their physical structure extremely fragile.

In other words, if the physical shockwave energy were strong enough to directly vibrate the lung parenchyma and irritate deep pulmonary vagal sensory nerve endings to trigger an autonomous respiratory dry cough, the prerequisite must be that the energy caused overt iatrogenic barotrauma. Clinically, this would inevitably be accompanied by acute pneumothorax, focal lung contusion, hemoptysis, and continuously worsening severe chest pain.

However, in actual clinical observation, all patients who experience stimulus-induced dry cough during procedures near C7 exhibit an absolutely “transient” feature that “stops immediately when the applicator is moved away”. Post-treatment, patients show no signs of dyspnea, decreased oxygen saturation, or bloody sputum, and auscultation and imaging examinations completely rule out organic changes such as pneumothorax. This significant clinical empirical evidence overturns the “physical lung contact theory” from the perspective of destructive mechanics.

## 3 Mechanism reconstruction: retrograde accessory nerve conduction and the “accessory-vagal crosstalk” hypothesis within the jugular foramen

After completely negating the traditional physical acoustics “lung vibration theory,” introducing modern precise neuroanatomical evidence is the cornerstone for accurately reconstructing this reflex arc. Anatomical studies by Bae et al. (7) regarding the distribution of the accessory nerve in the upper trapezius confirm that the main trunk of the accessory nerve (CN XI) innervating the upper trapezius and its dense motor endplate branches have a highly predictable and dense distribution from the level of the C7 spinous process to the vicinity of the Dazhui acupoint (Figure 1). The reference-line method used to divide the upper trapezius into four anatomical regions is illustrated in (Figure 2), while the dense arborization pattern of the accessory nerve is shown in (Figure 3).

**Figure 1:**
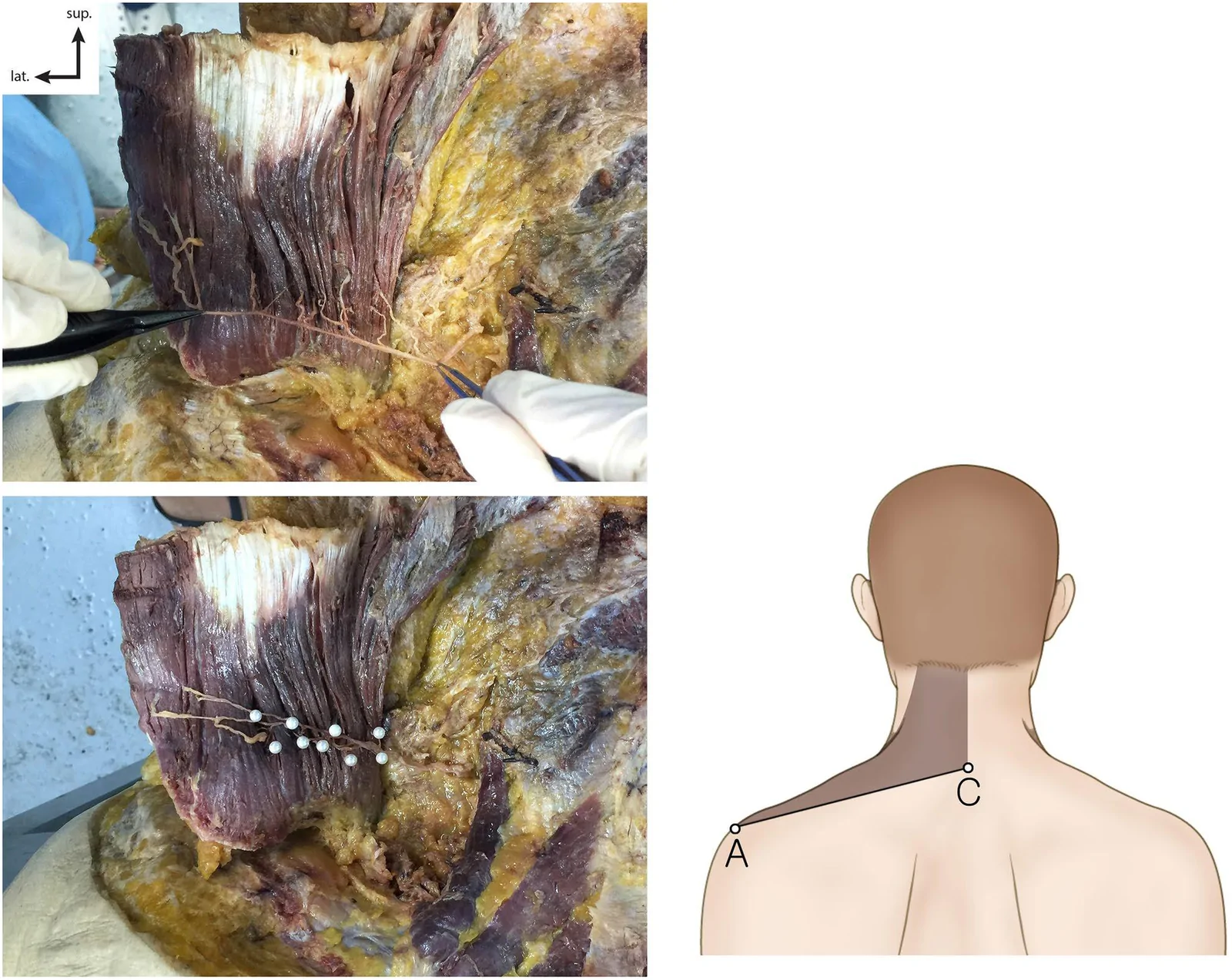
Photograph of the dissection of perforating points of the accessory nerve. White pins indicate perforating branches of the accessory nerve in the upper trapezius. Reproduced with permission from “Photograph of the dissection of perforating points of the accessory nerve” by Jung-Hee Bae, Ji-Soo Lee, Da-Yae Choi, JeongHoon Suhk and Seong Taek Kim.

**Figure 2:**
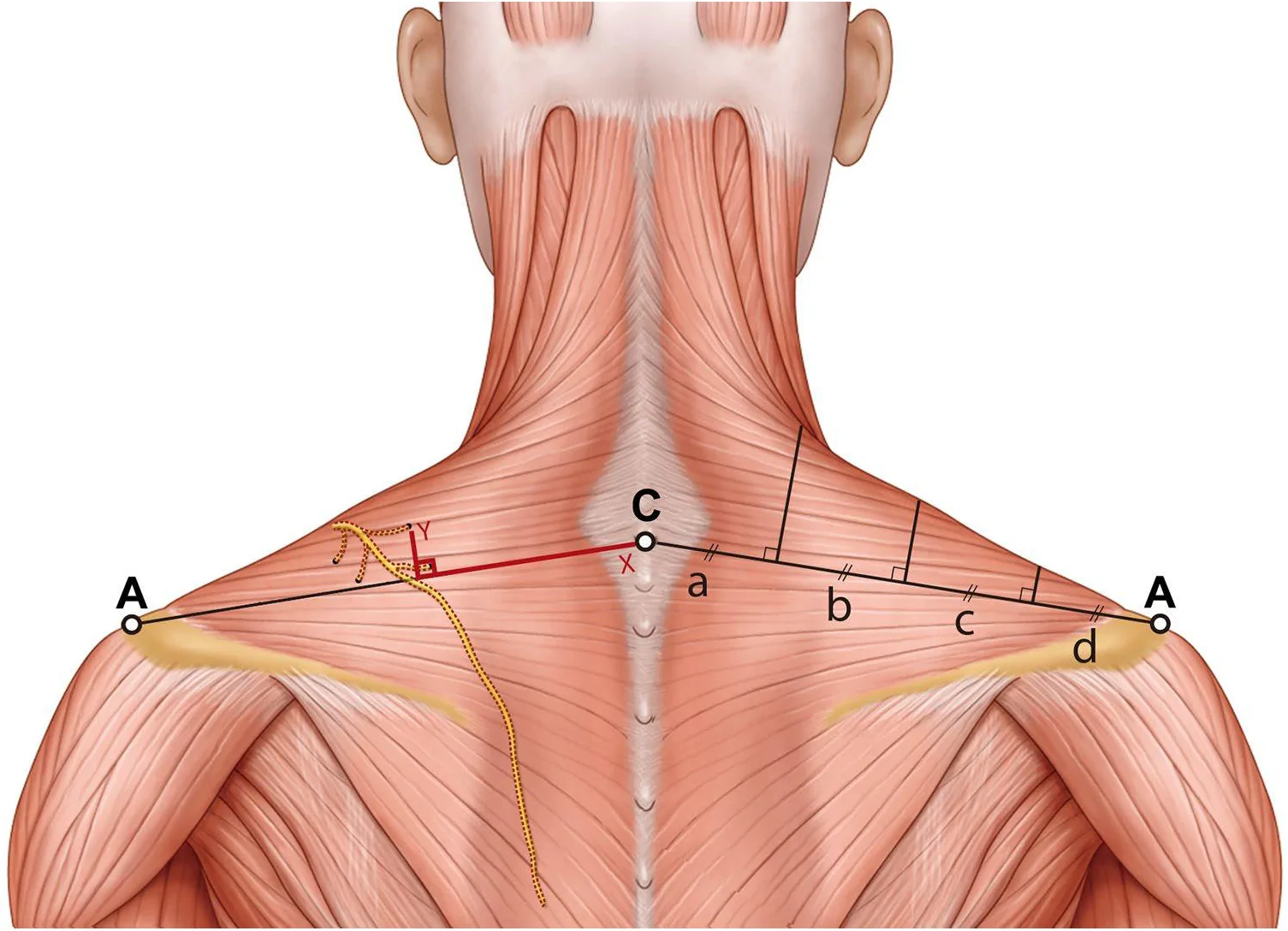
Illustration of the reference points and lines used to accessory nerve. C describes the distribution of the, 7th spinous process of the vertebra prominens; A, acromion; a–d, the line between C and A was divided into four equally sized sections; Yellow lines, schematization of perforating of the accessory nerve in the upper trapezius. Reproduced with permission from “Illustration of the reference points and lines used to describe the distribution of the accessory nerve” by Jung-Hee Bae, Ji-Soo Lee, Da-Yae Choi, JeongHoon Suhk and Seong Taek Kim.

**Figure 3:**
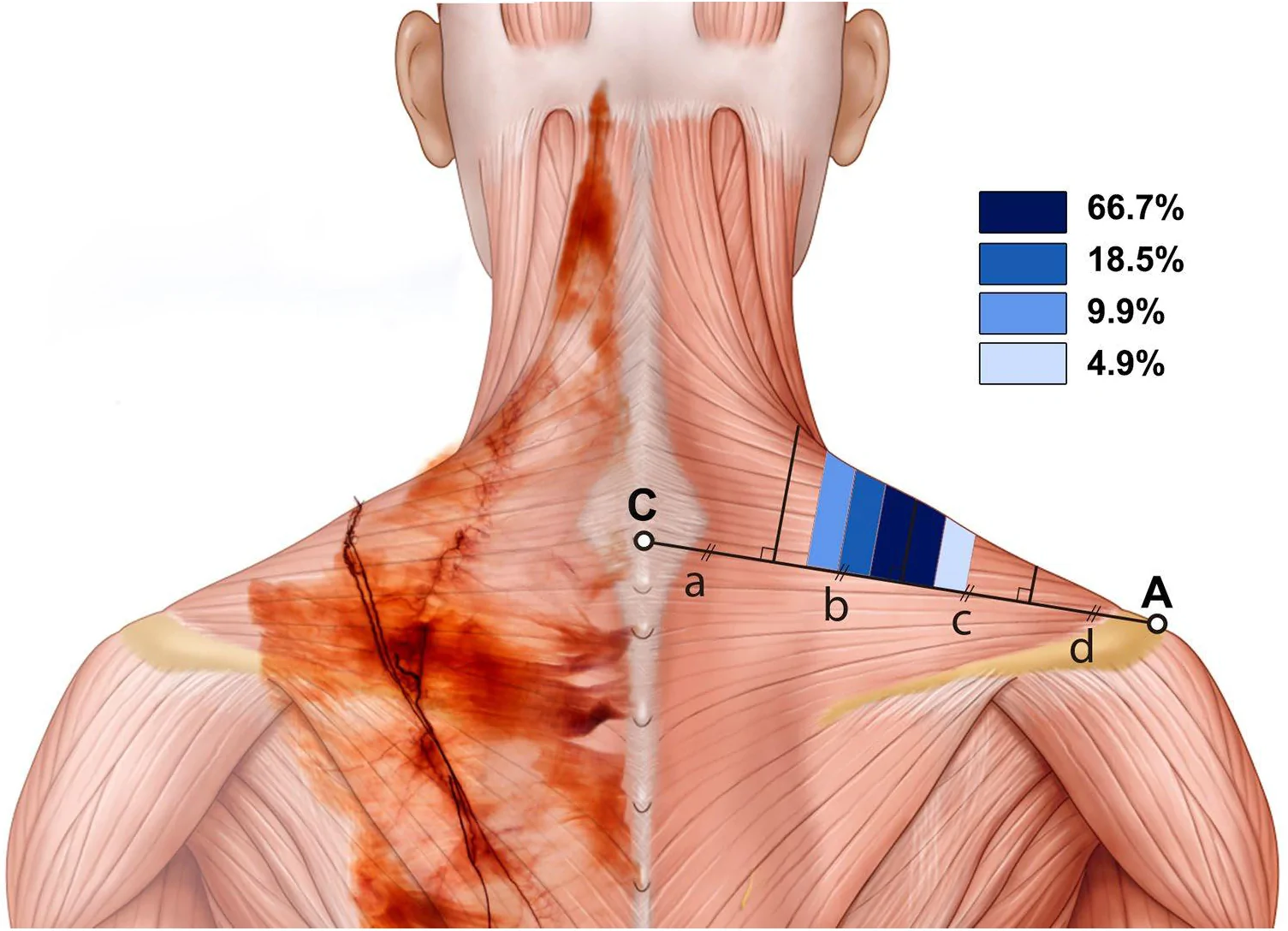
Left, photograph of accesory nerve; right, illustration of the dense arborization of the perforating accessory nerve branches. Blue areas, distribution of perforating points. Reproduced with permission from “Left, photograph of Sihler's staining of the accessory nerve; Right, illustration of the dense arborization of the perforating accessory nerve branches” by Jung-Hee Bae, Ji-Soo Lee, Da-Yae Choi, JeongHoon Suhk and Seong Taek Kim.

Based on this decisive anatomical discovery, we formally propose a novel cross-system conduction mechanism: the “Accessory-Vagal Crosstalk at the Jugular Foramen” hypothesis. This mechanism indicates that the stimulus-induced dry cough triggered by ESWT does not originate from local mechanical impact within the thoracic cavity. Instead, it is due to retrograde discharging of the accessory nerve after strong irritation, which triggers an “electrical potential spillover” at the jugular foramen of the skull base, thereby sounding the vagal cough reflex alarm.

- Shockwave vibration at the C7/Dazhui area.
- Strongly irritates the superficial main trunk of the upper trapezius.
- Detonates an explosive action potential in the Accessory Nerve (CN XI).
- Retrograde axonal conduction ascends across the neck.
- Reaches the Jugular Foramen at the cranial base.
- Ephaptic crosstalk occurs with the Vagus Nerve (CN X) and the Glossopharyngeal Nerve (CN IX).
- The signal ascends into the brainstem.
- Reaches the Nucleus Tractus Solitarius (NTS).
- Central misinterpretation triggers a defense command, resulting in an explosive irritative dry cough.

The proposed neurophysiological pathway is summarized in Figure 4.

**Figure 4:**
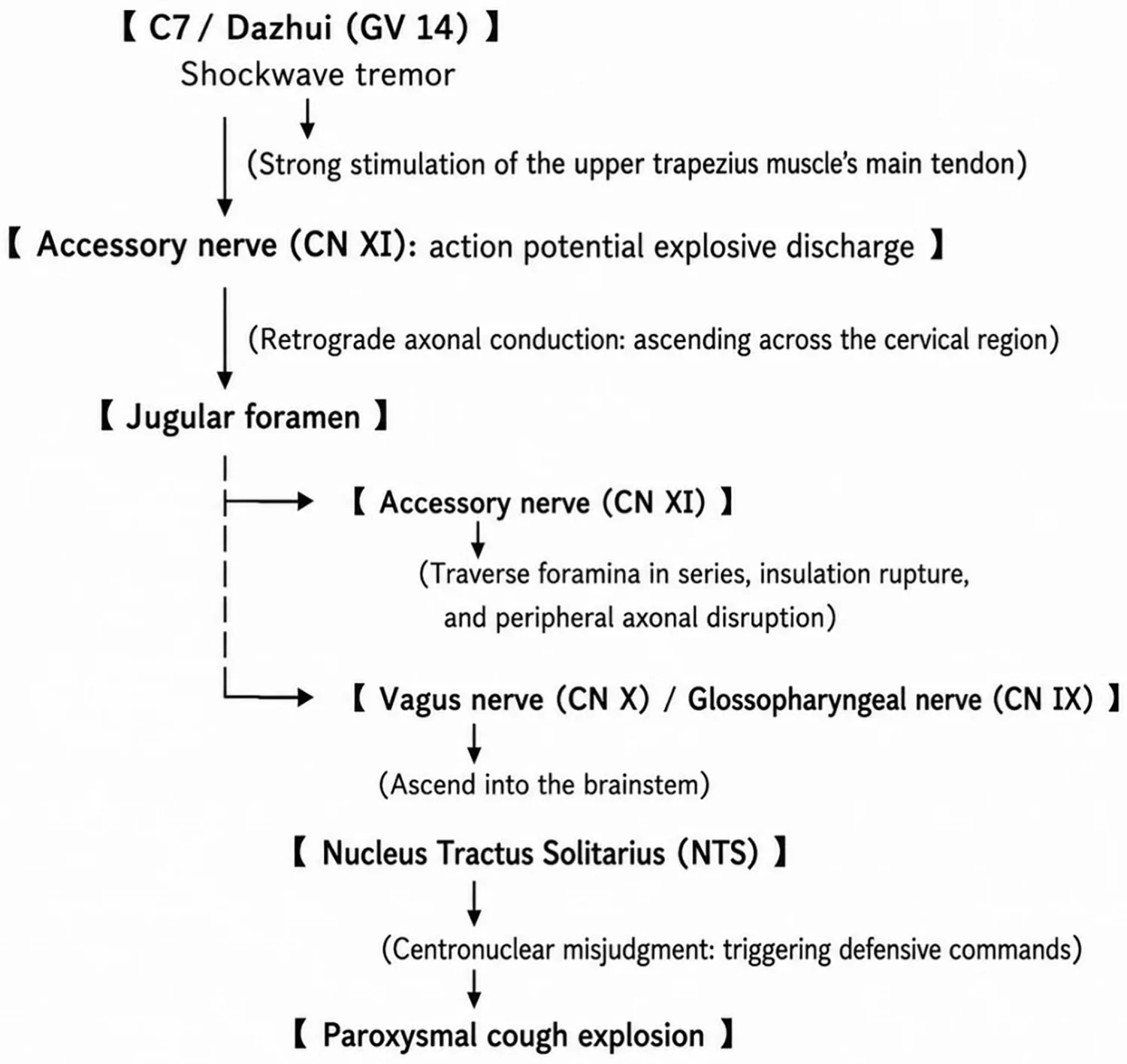
Proposed mechanism of accessory–Vagus nerve crosstalk at the jugular foramen.

### 3.1 Peripheral initiation: mechanical-electrical transduction burst of the accessory nerve near C7

As a special visceral motor nerve innervating the trapezius and sternocleidomastoid muscles, the superficial main trunk pathway of the accessory nerve (CN XI) after crossing the posterior cervical triangle lies precisely within the deep fascia layer of the upper trapezius. According to precise anatomical measurements, within a specific area extending laterally from the C7 spinous process level, the branches of the accessory nerve present a high-density network distribution; the nerve trunk is superficially located and highly susceptible to external physical stimulation (7).

When ESWT releases megapascal-level positive high pressure and broadband mechanical shear stress bombarding the C7 target, this superficially distributed accessory nerve trunk undergoes severe physical deformation. Because the energy density and displacement frequency of the shockwave far exceed daily physiological limits, High-intensity mechanical stimulation is known to activate mechanically gated ion channels, particularly members of the PIEZO family, thereby converting mechanical force into electrical activity (8). This leads to a massive Na + influx, instantly detonating extremely high-amplitude, high-frequency, and highly synchronized action potential clusters retrogradely (antidromic conduction) at the periphery (9). This may induce antidromic propagation of action potentials along the accessory nerve, a phenomenon that has been well documented in peripheral nerves.

### 3.2 Anatomical bottleneck: high nerve concentration within the jugular foramen

After the accessory nerve fibers are irritated, action potentials travel rapidly backward along the nerve trunk toward the skull base. The anatomical bottleneck they must pass through is the Jugular Foramen. In skull base anatomy, the Jugular Foramen is an extremely narrow bony canal with a highly congested internal space. Within this tiny bony passage, three crucial cranial nerves run side-by-side: the glossopharyngeal nerve (CN IX), the vagus nerve (CN X), and the accessory nerve (CN XI). These three cranial nerves are not only extremely close spatially within the Jugular Foramen (usually separated only by micrometer-level connective tissue sheaths) but also share the same dural envelope. This makes the Jugular Foramen a natural “electrically non-insulated area for nerves”. The anatomical relationship among cranial nerves IX, X, and XI within the jugular foramen is illustrated in (Figure 5), providing the structural basis for the proposed ephaptic crosstalk mechanism.

**Figure 5:**
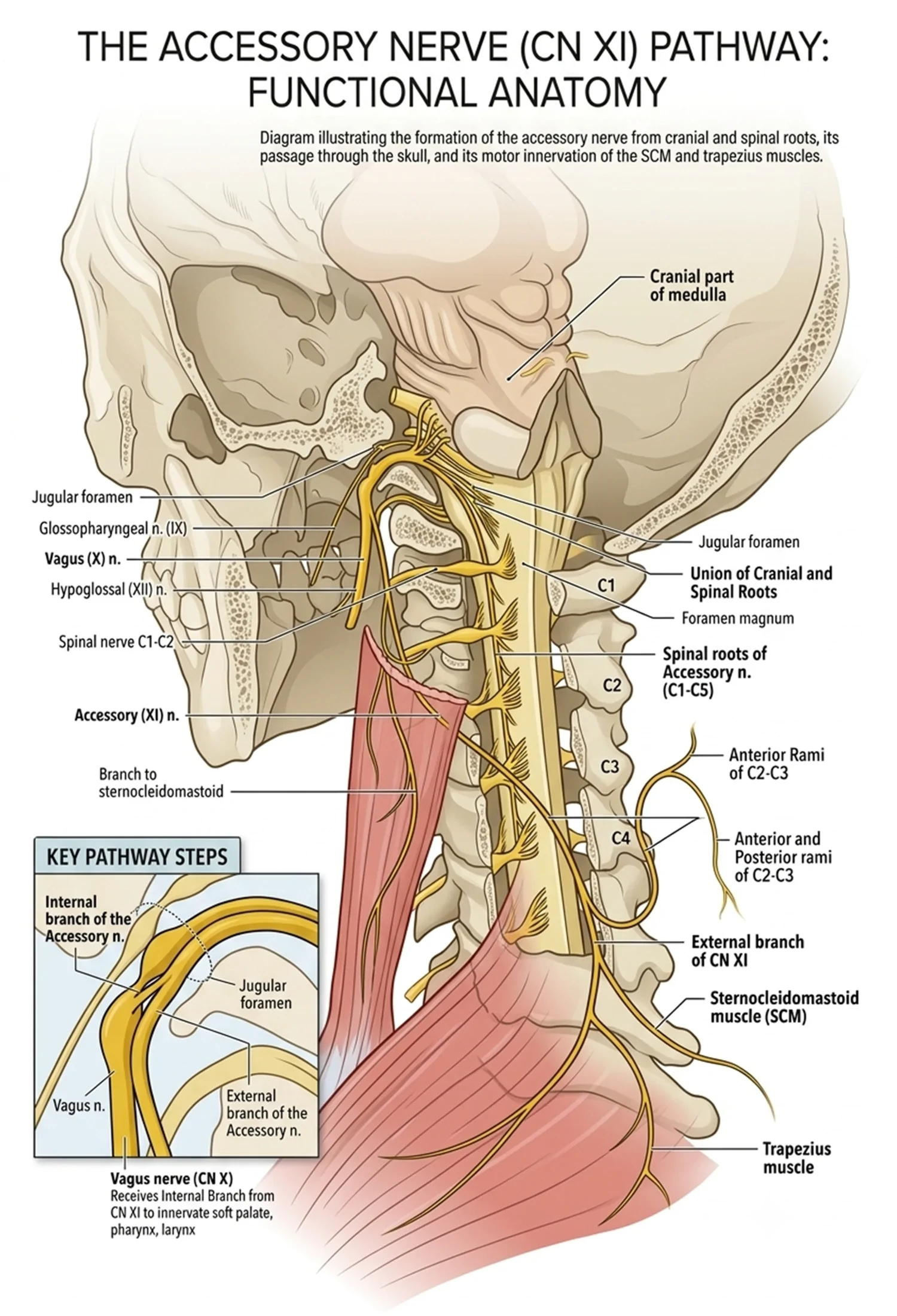
The accessory nerve (CN XI) pathway. Educational anatomical illustration demonstrating the cranial and spinal roots of the accessory nerve (CN XI), their course through the foramen magnum and Jugular Foramen, and their motor innervation of the sternocleidomastoid and trapezius muscles.

### 3.3 Peripheral ephaptic crosstalk and nerve electrical spillover

We hypothesize that high-frequency mechanical stimulation of the accessory nerve may generate antidromic electrical activity that propagates proximally toward the skull base. Within the confined anatomical space of the jugular foramen, where the glossopharyngeal (CN IX), vagus (CN X), and accessory (CN XI) nerves course in close proximity, such abnormal neural activity could theoretically increase the likelihood of nonsynaptic (ephaptic) interactions between adjacent nerve fibers. Although direct electrophysiological evidence for this phenomenon in the jugular foramen is currently lacking, ephaptic transmission has been demonstrated in other regions of the nervous system under conditions of close axonal apposition and altered extracellular electrical fields (10).

Based on this concept, we further hypothesize that aberrant activation of nearby vagal sensory fibers could occur without direct stimulation of the airway. Because vagal afferents normally convey sensory information from the respiratory tract to the nucleus tractus solitarius (NTS), such ectopic activation may be interpreted by the brainstem as airway-related sensory input, thereby initiating a cough reflex despite the absence of a physical airway stimulus (11). This proposed mechanism remains speculative and requires future electrophysiological and neuroanatomical validation.

### 3.4 Brainstem terminal interpretation: passive activation of the NTS and pseudo-defense

The vagal sensory electrical signals mistakenly activated via Jugular Foramen crosstalk subsequently project upward along the vagus nerve trunk into the core control console of the brainstem—the nucleus tractus solitarius (NTS) in the medulla (12).

The NTS is the highest lower-level command center for the human respiratory defense mechanism. Under normal conditions, signals are transmitted to the NTS via the vagus nerve to trigger a cough only when cough receptors in the tracheal mucosa are subjected to foreign bodies or chemical stimulation. However, when “crosstalk signals” from the Jugular Foramen flood into the NTS, secondary sensory neurons within the NTS cannot distinguish whether this signal originates from a “true respiratory tract invasion” or “leakage conduction from the accessory nerve at the skull base”. Out of an instinctive survival protection mechanism, the NTS directly judges it as the highest-level airway obstruction threat. The central command center instantly overloads and issues an explosive defense command: through the descending nucleus ambiguus and phrenic nerve nucleus, it drives the recurrent laryngeal nerve, phrenic nerve, and intercostal nerves to discharge synchronously and explosively, coordinating severe contractions of the diaphragm, abdominal muscles, and laryngeal muscles—ultimately resulting in the patient erupting into an uncontrolled, irritative dry cough the moment the shockwave hits C7.

### 3.5 Mechanism: closed-loop: threshold breakthrough in cough hypersensitivity syndrome (CHS)

Clinically, not all patients receiving C7 shockwave therapy experience coughing, which highly suggests the existence of individual threshold differences. This phenomenon can be explained by Cough Hypersensitivity Syndrome (CHS) (13). For patients suffering long-term from chronic pharyngitis, gastroesophageal reflux disease (GERD), allergic rhinitis, or those who have recently had a cold, the microenvironment within their NTS is inherently in a highly irritated state of central sensitization. The neuronal firing threshold in this highly sensitive population is extremely low, such that faint retrograde “leakage” from the accessory nerve is sufficient to cross their fragile synaptic threshold and instantly detonate the entire reflex arc.

## 4 Clinical implications and operational guidelines

While the “Accessory-Vagal Crosstalk at the Jugular Foramen” hypothesis provides a compelling theoretical framework for the observed phenomenon, clinical protocols must remain grounded in empirical, evidence-based outcomes. The primary clinical objective is to safely manage the elicited symptoms and accurately differentiate them from severe iatrogenic adverse events.

### 4.1 Observational characteristics and clinical differentiation

When applying ESWT near the C7 region, practitioners must carefully differentiate between a benign, transient reflex cough and potential iatrogenic pulmonary injury, such as pneumothorax or barotrauma. Clinical observations indicate that the reflex cough exhibits a distinct “Instantaneous On-Off Effect,” which serves as the primary diagnostic differentiator (Table 1).

**Table 1:** Comparison of mechanism, symptoms, and management between ESWT-induced dry cough and others.

Differential Point	Transient Reflex Dry Cough	Suspected Pulmonary Injury
Onset Timing	Occurs instantaneously upon shockwave irritation of the specific target.	Progressively worsens during or after treatment.
Shutdown Reaction	Abrupt cessation within milliseconds once the applicator is moved or turned off.	Cough persists and typically worsens despite cessation of therapy.
Accompanying Signs	No respiratory distress; normal oxygen saturation (SpO2).	Chest tightness, shortness of breath, cyanosis, and progressive decline in SpO2.
Chest Auscultation	Clear, symmetrical breath sounds bilaterally.	Diminished or absent breath sounds on the affected side; moist rales may be present.
Clinical Management	Proceed with treatment after fine-tuning the applicator angle or lowering energy.	Terminate treatment immediately; initiate emergency respiratory evaluation.

### 4.2 Evidence-informed operational adjustments

Although the exact cross-system neural pathways require further electrophysiological validation, empirical clinical practice has identified several operational adjustments that effectively mitigate the occurrence of the stimulus-induced cough while preserving therapeutic efficacy.

- **Pre-treatment Patient Screening:** Patients with a history of airway hyperresponsiveness—including chronic pharyngitis, gastroesophageal reflux disease (GERD), allergic rhinitis, or recent respiratory infections—appear clinically more susceptible to this reflex (13). Routine pre-treatment screening and providing targeted informed consent regarding the potential for a transient dry cough are recommended for these sensitive populations.
- **Applicator Angulation:** Clinical experience suggests that avoiding a direct, perpendicular application over the posterior cervical skin reduces the incidence of coughing. Adjusting the applicator to a 15° to 30° tilt, directing the acoustic field obliquely toward the cervical vertebral body, effectively bypasses direct superficial irritation while maintaining therapeutic targeting of the deep myofascial layers.
- **Stepwise Energy Titration:** Initiating treatment with high energy is clinically counterproductive in this anatomical region. Employing a “low-frequency initiation, stepwise titration” strategy—such as starting at 4 to 6 Hz with low pressure and gradually increasing the intensity—allows for patient tolerance assessment and minimizes the sudden elicitation of the reflex cough.

## 5 Limitations and future directions

Although the “Accessory-Vagal Crosstalk at the Jugular Foramen” hypothesis provides a logically rigorous cross-system explanatory loop for ESWT-induced irritative dry cough through cross-validation of modern neuroanatomy and clinical physical acoustics, as a pioneering theoretical exploration, this hypothesis still has certain limitations at the current stage and needs further confirmation in future basic and clinical research.

### 5.1 Main limitations of this hypothesis

**Lack of direct *in vivo* electrophysiological evidence:** The core mechanism of this hypothesis—"axonal leakage and ephaptic transmission” within the jugular foramen (10)—is currently deduced backward primarily based on spatial position measurements from cadaveric dissections (the micrometer-level proximity of CN IX, CN X, and CN XI) (14) and the clinical “on-off effect” during episodes. Constrained by the bottlenecks of current non-invasive medical detection technologies, it is currently impossible to directly implant microelectrodes in living humans to synchronously capture local extracellular microelectric field changes and passive depolarization potentials of the vagus nerve induced in the jugular foramen when the accessory nerve is strongly vibrated.

**Insufficient quantitative consideration of microanatomical variations in bony canals:** The hypothesis mentions that central sensitization (CHS) is one of the causes of individual susceptibility but fails to fully quantify the impact of cranial base anatomical variations. Significant individual differences exist in the morphology, pore size, and thickness of internal connective tissue septa of the jugular foramen among the population. How these physical structural variations specifically affect the “electrical insulation strength” between nerves and their impact on the threshold of crosstalk currently lacks precise imaging anatomical calculations.

### 5.2 Future research directions

To confirm and refine this theoretical model, future cross-disciplinary research can be conducted along the following three core dimensions:

***In vivo* Electrophysiological Mapping Based on Animal Models:** *in vivo* experimental models can be constructed using large mammals (such as minipigs or macaques, whose cervical anatomy and skull base canal characteristics are highly homologous to humans). While simulating clinical ESWT doses to mechanically bombard the superficial layer of the upper trapezius, multi-electrode arrays can be utilized to synchronously record the retrograde firing rate of the accessory nerve trunk, the afferent impulses of the vagus nerve, and the firing rate activity of neurons in the nucleus tractus solitarius (NTS) in the medulla. This will provide the most direct electrophysiological evidence for the full chain of “mechanical wave-peripheral potential crosstalk-central misdecoding”.

**Neuroimaging and Brainstem Functional Mapping:** At the human clinical research level, functional magnetic resonance imaging (fMRI) or positron emission tomography (PET-CT) can be introduced. When subjects receive a modified low-frequency localized mechanical vibration (simulating shockwave irritation), the instantaneous enhancement of blood oxygen level-dependent (BOLD) signals in the brainstem, particularly in the NTS and nucleus ambiguus regions, can be monitored in real time, thereby providing visual imaging support for central convergence and the issuance of pseudo-defense commands under non-invasive conditions.

**Large-scale Randomized Controlled Trials (RCTs) for Modified Physical Operation Protocols:** Based on the cough prevention operation guidelines derived from this hypothesis—"fine-tuning the applicator angle (15° to 30° oblique)” and “low-energy stepwise titration"—multicenter, large-sample randomized controlled trials (RCTs) should be conducted in the future. By quantitatively comparing the statistical differences between traditional vertical targeted irradiation and modified oblique minimally invasive operations in “iatrogenic dry cough induction rate,” “Visual Analog Scale (VAS) for pain/discomfort during treatment,” and “long-term myofascial functional recovery (NDI index),” the clinical translational value of this hypothesis can be inversely verified, promoting the formation of a national expert consensus on safe ESWT operations in orthopedics/rehabilitation departments.

## Data Availability

The original contributions presented in the study are included in the article/Supplementary Material, further inquiries can be directed to the corresponding author.
